# Visceral Fat Area and Serum Adiponectin Level Predict the Development of Metabolic Syndrome in a Community-Based Asymptomatic Population

**DOI:** 10.1371/journal.pone.0169289

**Published:** 2017-01-03

**Authors:** Sang-A Cho, Hyung Joon Joo, Jae-Young Cho, Seung Hun Lee, Jae Hyoung Park, Soon Jun Hong, Cheol Woong Yu, Do-Sun Lim

**Affiliations:** Department of Cardiology, Cardiovascular Center, Korea University Anam Hospital, Seoul, Korea; National Taiwan University College of Medicine, TAIWAN

## Abstract

**Background:**

Although it has been demonstrated that visceral adipose tissue content and serum levels of adiponectin are associated with metabolic syndrome, their predictive potential for the development of metabolic syndrome remains to be elucidated.

**Methods:**

We studied 1,130 participants of the Seoul Metabolic Syndrome cohort. A total of 337 subjects without metabolic syndrome underwent the follow-up evaluation and finally analyzed. Visceral fat area (VFA) was measured using dual bioelectrical impedance analysis. We compared the 1-year incidence rate of metabolic syndrome among four different groups: Group 1 (high adiponectin level and low VFA), Group 2 (low adiponectin level and low VFA), Group 3 (high adiponectin level and high VFA) and Group 4 (low adiponectin level and high VFA).

**Results:**

Median follow-up duration was 17 months. Cut-off points of adiponectin level and VFA for metabolic syndrome were 7.34 ng/ml and 84 cm^2^ for men, and 12.55 and 58 cm^2^ ng/ml for women, respectively. The incidence of metabolic syndrome was the highest in Group 4 (Group 1; 16.47%, Group 2; 22.08%, Group 3; 25%, and Group 4; 46.15%, p<0.001). Adjusted logistic regression analyses for metabolic syndrome prediction demonstrated that Group 4 exhibited the highest odds ratio compared with Group 1 (4.918 [2.05–11.795]), which was predominantly affected by waist circumference and serum triglyceride levels. Notably, triglyceride/high-density lipoprotein cholesterol (TG/HDL) ratio was significantly higher in Group 4 (p = 0.017).

**Conclusion:**

Incidence rate of metabolic syndrome was the highest in subjects with low serum adiponectin levels and high visceral fat area. Higher TG/HDL ratio in these subjects suggested insulin resistance may contribute to the development of metabolic syndrome.

## Background

Prevalence of metabolic syndrome, which is composed of abdominal obesity, high blood pressure, hyperglycemia, and dyslipidemia, is increasing worldwide and is known to cause cardiovascular diseases [1–3]. From this perspective, it is essential that risk factors that cause metabolic syndrome are elucidated.

Obesity has long been studied with metabolic syndrome, and it is associated with adiposity and insulin resistance. Insulin resistance is an important factor that can predict the cause of metabolic syndrome. Several studies have shown that visceral adipose tissue pertaining to insulin resistance is correlated with obesity-related disease [4–8]. Visceral adipose tissue is also associated with metabolic syndrome [9–11].

There were also several longitudinal studies on the impact of visceral adiposity on metabolic syndrome. The MESA study showed that visceral adiposity significantly increased the metabolic syndrome risk during the median 3.3-year follow-up period [12]. The Hitachi Health Study revealed that more than 50 cm2 increase in visceral fat area during 3-year follow-up period was significantly associated with the incidence of metabolic risk factors, especially high triglyceride and low high-density lipoprotein levels [13]. These suggested that visceral adipose tissue might play an important role in the progression to metabolic syndrome.

Previous studies have suggested that serum levels of adiponectin are associated with metabolic syndrome [14–19] and metabolic syndrome development may be associated with obesity, adipose tissue content, and hormonal levels. Adipokines, such as adiponectin, are secreted by the adipose tissue or as a result of glucose and lipid metabolism.[20–22]. Accordingly, the level of adiponectin is associated with obesity-related disorders and metabolic risk factors [23–26]. Adiponectin levels are inversely correlated with visceral obesity; therefore, high levels of adiponectin are negative correlated with obesity whereas low adiponectin levels exhibit a positive correlation^9^.

Although visceral adipose tissue and serum levels of adiponectin have been associated with metabolic syndrome, their predictive potential for the development of metabolic syndrome remains unknown. The present study aimed to investigate the predictability of serum adiponectin levels and visceral adipose tissue for the development of metabolic syndrome.

## Methods

### Study population

This is a prospective cohort study of the Seoul Metabolic Syndrome (S-MAS) study researched by Korea University Anam hospital in Seoul, Korea. 1,130 participants of both sexes between 30 and 64 years of age suspected of having metabolic syndrome were enrolled from 25 public healthcare centers from January 2014 to September 2014. Subjects with a previous history of angina pectoris, myocardial infarction, stroke, or any revascularization were excluded for the enrollment. At each visit, demographic characteristics including age, underlying diseases and medications were collected through a standardized questionnaire. Basic medical examinations, including a physical examination, were performed by physicians. Abdominal fat content measurements and other laboratory examinations were also performed. From May 2015 to November 2015, 756 participants performed the follow-up evaluation. The follow-up loss rate was 33.1% (n = 374). Among the remaining 756 participants, 419 subjects was excluded due to the following reasons: metabolic syndrome at baseline (n = 258), missing serum adiponectin levels (n = 84), missing visceral adipose tissue area (n = 179), in addition to being treated with antihypertensive medication (n = 67), antidiabetic medication (n = 17), and antihyperlipidemic medication (n = 63). 249 subjects had concurrently two reasons or more than two. The present study was approved by the institutional review board of Korea University Anam Hospital (IRB NO. ED13087) and performed in accordance with the principles of the Declaration of Helsinki. Written informed consent was obtained.

### Abdominal fat content measurement

Abdominal fat content was measured by a trained nurse by using the Visceral Fat scan device (Omron HDS-2000) according to the manufacturer’s protocol. Briefly, visceral fat area (VFA) and subcutaneous fat area (SFA) were measured at the umbilicus level according to the dual bioelectrical impedance analysis method. The correlation between abdominal fat areas measured by this method and computed tomography was good, with a correlation coefficient of 0.888 [27].

### Laboratory tests

Blood samples were obtained from each subject after at least 8 hours of fasting. Serum glucose levels were determined using a UV assay. Serum levels of total cholesterol, low density lipoprotein (LDL)-cholesterol, high density lipoprotein (HDL)-cholesterol and triglycerides were measured using the homogeneous enzymatic colorimetric assay. Serum adiponectin levels, high-sensitivity C-reactive protein (hsCRP), apolipoprotein A1, and apolipoprotein B were gauged from the immunoturbidimetry assay.

### Definitions

**Fig 1:**
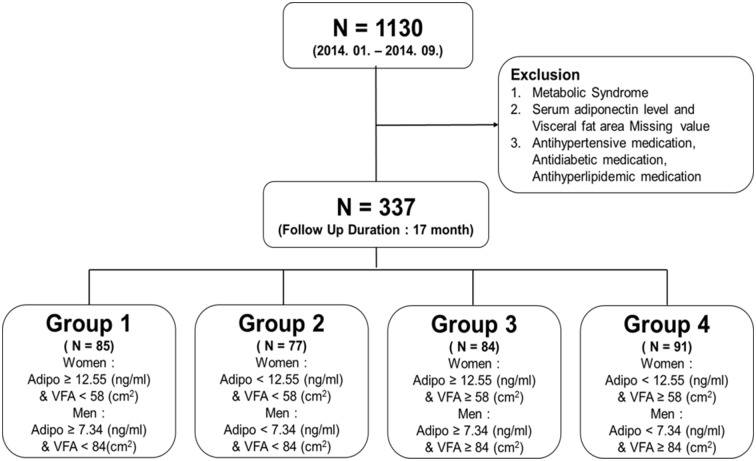
Study population. Adipo, Serum adiponectin level; VFA, Visceral fat area.

Subjects were categorized into four groups according to the combinations of the median of VFA and serum adiponectin levels for each sex. The median serum adiponectin level was 9.83 ng/mL in the total population, 12.55 ng/mL in women, and 7.34 ng/mL in men. The median of VFA was 68 cm^2^ in the total population, 58 cm^2^ in women, and 84 cm^2^ in men. The definition of the four groups was as follows (Fig 1): i) adiponectin level ≥ 12.55 ng/mL and VFA < 58 cm^2^ for women and adiponectin level ≥ 7.34 ng/mL and VFA < 84 cm^2^ for men (Group 1); ii) adiponectin level < 12.55 ng/mL and VFA < 58 cm^2^ for women and adiponectin level < 7.34 ng/mL and VFA < 84 cm^2^ for men (Group 2); iii) adiponectin level ≥ 12.55 ng/ml and VFA ≥ 58 cm^2^ for women and adiponectin level ≥ 7.34 ng/ml and VFA ≥ 84 cm^2^ for men (Group 3); and iv) adiponectin level < 12.55 ng/mL and VFA ≥ 58 cm^2^ for women and adiponectin level < 7.34 ng/mL and VFA ≥ 84 cm^2^ for men (Group 4).

Metabolic syndrome was defined according to the National Cholesterol Education Program Expert Panel on Detection, Evaluation, and Treatment of High Blood Cholesterol in Adults, Adult Treatment Panel III and the Korean Society for the Study of Obesity. Therefore, a person who exhibited any at least three of the following five factors was considered to have metabolic syndrome: i) waist circumference ≥90 cm for men and ≥80 cm for women; ii) blood pressure ≥ 130/85 mmHg; iii) fasting glucose level ≥ 100 mg/dL; iv) triglycerides level ≥150 mg/dL; and v) HDL cholesterol level <40 mg/dL for men and <50 mg/dL for women.

### Statistical analysis

Continuous variables were presented as mean ± standard deviation or median (25th percentile, 75^th^ percentile). Categorical variables were presented as counts (percentages) and were analyzed by chi-square test and Fisher’s exact test for group comparisons. Normality was examined by the Kolmogorov-Smirnov test for the analysis of each variable. For continuous variables, the comparison of four groups was performed using one way analysis of variance for normal distributions or by the Kruskal-Wallis test for non-normal distributions. In addition, post-hoc Bonferroni contrast analysis was performed.

The logistic regression model was used to elucidate the association between four groups or the quartile of each factor (adiponectin level and VFA) and metabolic syndrome or five components of it. Associations were presented as an odds ratio (OR) with 95% confidence intervals (CI). In order to investigate the association, Model 1 was adjusted for age and sex. Model 2 was adjusted for Model 1 adjusted factors, smoking status, alcohol intake, education level, and physical activity. Model 3 was further adjusted for five continuous components of metabolic syndrome. Additionally, Model 4 was adjusted for hsCRP, LDL-cholesterol, apolipoprotein A1 and apolipoprotein B. A two-sided p-value of less than 0.05 was considered statistically significant. All statistical analyses were performed using SAS 9.3 (SAS Institute Inc., Cary, NC, USA) software.

## Results

### Baseline characteristics

The association between the level of adiponectin and VFA exhibited a negative correlation (r = -0.1371, p <.0001). The initial demographic features are presented in Table 1. Study subjects consisted of 189 women (56.08%) and 148 men (43.92%). The median age of subjects was 56. In Group 1 (n = 85), there were 34 (40%) men with a median age of 56. Group 2 (n = 77) consisted of 38 (49.35%) men, with a median age of 56. Group 3 (n = 84) was composed of 40 (47.62%) men, and subjects were median aged 58 years. In Group 4 (n = 91), there were 36 (39.56%) men aged 56 years. Body mass index (BMI) and waist circumference were demonstrated to be significantly different among the four groups (p <.001). These factors were increased in Groups 3 and 4, as compared with Groups 1 and 2. Laboratory findings demonstrated that triglyceride levels were increased in Group 4 and high-density lipoprotein (HDL) cholesterol levels were increased in Group 1. Therefore, the triglyceride to HDL-cholesterol ratio was significantly different among the groups (p = 0.017) and was the highest in Group 4. Total cholesterol, fasting glucose, LDL-cholesterol levels and blood pressure were similar among the groups.

**Table 1:** Baseline characteristics.

Variable	Group 1 (N = 85)	Group 2 (N = 77)	Group 3 (N = 84)	Group 4 (N = 91)	p-value
Adiponectin (ng/mL) ₀	17.07±8.03a	6.80±3.10b	15.37±5.01a	6.38±2.90b	<.001
Visceral fat area (cm^2^) ₀	50(38,64)a	55(45,69)a	91(66,108)b	87(69,102)b	<.001
Men (%)	34(40)	38(49.35)	40(47.62)	36(39.56)	0.454
Age (years)	56(52,60)	56(51,59)	58(54,61)	56(52,60)	0.101
Waist circumference (cm) ₀	82.11±8.00a	82.87±5.94a	88.8±7.91b	88.35±6.79b	<.001
BMI (kg/m^2^) ₀	23.85±2.67a	23.88±2.13a	26.05±2.61b	26.37±2.63b	<.001
Current Smoker (%)	16(19.05)	15(19.48)	13(15.48)	13(14.44)	0.767
Systolic blood pressure (mmHg)	118.65±13.34	117.95±13.21	118.16±12.34	119.86±12.72	0.768
Diastolic blood pressure (mmHg)	73.71±9.17	74.29±9.15	73.17±9.46	75.03±9.5	0.594
Pulse pressure (mmHg)	72(66,79.5)	70.5(66,78.5)	69(62.5,77.5)	72(66.5,81)	0.263
Total Cholesterol (mg/dL)	201.92±32.36	197.29±32.63	205.83±32.09	202.79±34.34	0.429
LDL-cholesterol (mg/dL)	133.91±28.21	132.95±31.99	138.86±29.37	136.74±32.37	0.590
HDL-cholesterol (mg/dL) ₀	57.66±13.64a	51.27±11.97b	53.94±11.46a,b	52.7±10.97b	0.006
Triglyceride (mg/dL) ₀	111(76,138)a	108(85,130)a	116(92.5,138)a,b	124(98,152)b	0.020
TG / HDL ratio ₀	1.88(1.24,2.69)a	2.2(1.5,2.89)a,b	2.24(1.76,2.84)b	2.4(1.75,3.16)b	0.017
Glucose (mg/dL)	93(88,100)	84(89,99)	94.5(90,100)	84(89,98)	0.462
High sensitivity CRP (mg/dL)	0.6(0.3,1.1)	0.4(0.3,1.0)	0.6(0.3,1.0)	0.7(0.4,1.4)	0.061
Apolipoprotein B (mg/dL)	98(85.4,114.1)	99.7(86.7,116.3)	101.85(90.35,116.7)	104(90,117.7)	0.352
Apolipoprotein AI (mg/dL)	143.3(128.9,162.7)	135(121.7,151.8)	143.05(128.6,156.65)	139.4(127.9,15.8)	0.086
Apolipoprotein B / A1 ratio	0.56(0.33,0.64)	0.73(0.60,0.88)	0.73(0.60,0.89)	0.76(0.63,0.86)	0.062

Data are presented as the mean ± standard deviation or (25 percentile, 75 percentile) for continuous variables and the number (%) for categorical variables.

BMI, body mass index; LDL, low-density lipoprotein; HDL, high-density lipoprotein; TG/HDL, Triglyceride/HDL-cholesterol; CRP, C-reactive protein.

^₀^ Multiple comparison (post-hoc) results, same letter means no difference.

### Incidence of metabolic syndrome and follow-up

**Fig 2:**
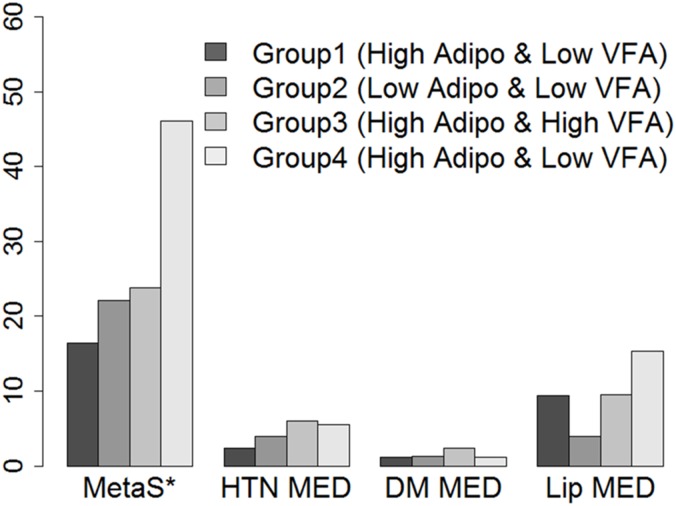
Incidence of metabolic syndrome at follow-up. Adipo, Serum adiponectin level; VFA, Visceral fat area; MetaS, Metabolic syndrome; HTN MED, Antihypertensive Medication; DM MED, Antidiabetic Medication; Lip MED, Antihyperlipidemic Medication. * Result of comparison between Groups, p <.0001.

During the median 17-month follow-up, metabolic syndrome was newly developed in 94 (27.89%) subjects. Incidence of metabolic syndrome was 16.47% in Group 1, 22.08% in Group 2, 25% in Group 3 and 46% in Group 4 (Group 4 vs other groups, p <.001) (Fig 2). However, the initiation rates of anti-hypertensive, anti-diabetic, and anti-hyperlipidemic medications were not significantly different among the groups.

Table 2 shows the follow-up data and their changes respective to the baseline. As compared with the baseline characteristics, follow-up waist circumference and BMI were significantly different (p <.001), and were demonstrated to be the highest in Groups 3 and 4. Triglyceride, HDL-cholesterol and the Triglyceride to HDL-cholesterol ratio also exhibited significant differences among the groups (all, p <.05). HDL cholesterol levels were the highest in Group1 and triglyceride levels and the Triglyceride to HDL-cholesterol ratio were lowest in Group 1 and highest in Group 4. Among these variables, only the alterations in triglyceride levels were significantly different among the groups (p = 0.042). Group 4 exhibited the largest increase in triglyceride levels between the baseline and follow-up. Notably, hsCRP levels at the follow-up were significantly different (p = 0.002) and were the highest in Group 4, although there were no significant differences detected at baseline (p = 0.061).

**Table 2:** Follow-up characteristics and their respective variations from the baseline.

Variable	Group 1 (N = 85)	Group 2 (N = 77)	Group 3 (N = 84)	Group 4 (N = 91)	p-value
Waist circumference (cm) ₀	84(80,90)a	85(81,89)a	89.5(85,94.5)b	90(87,93.5)b	<.001
Δ Waist circumference (cm)	1.5(-2,4)	1.5(0,5)	1(-1,3.5)	2(-1,5)	0.690
BMI (kg/m2) ₀	23.34(22.04,24.65)a	23.37(22.06,24.8)a	25.16(23.94,27.54)b	25.95(24.53,27.31)b	<.001
Δ BMI (kg/m2)	-0.34(-0.89,0.29)	-0.3(-0.97,0.32)	-0.32(-0.95,0.36)	-0.32(-0.98,0.32)	0.971
Systolic blood pressure (mmHg)	119.4±13.51	120.7±13.06	119.2±14.56	122.25±12.98	0.414
Δ Systolic blood pressure (mmHg)	0.83±13.65	2.75±12.67	1.63±12.77	2.4±12.47	0.780
Diastolic blood pressure (mmHg)	78.43±8.61	80.77±8.54	78.65±10.41	80.47±7.87	0.203
Δ Diastolic blood pressure (mmHg)	4.75±6.19	6.48±8.07	5.68±7.32	5.44±8.49	0.547
Pulse pressure (mmHg)	71.5(64,78.5)	72(66,84)	70.75(62,76.75)	71(65.5,79)	0.328
Δ Pulse pressure (mmHg)	-0.5(-7,7)	2.5(-3,7.5)	0(-6.5,6)	-1(-9,7)	0.189
Total Cholesterol (mg/dL)	206(186,235)	199(180,219)	200(183,226)	205(189,233)	0.371
Δ Total Cholesterol (mg/dL)	11(-13,28)	8(-9,27)	2(-19,15)	6(-10,30)	0.148
LDL-cholesterol (mg/dL)	140.07±33.35	135.1±32.07	136.25±28.78	139.29±35.78	0.727
Δ LDL-cholesterol (mg/dL)	10(-9,27)	3(-11,17)	1(-15,16)	2(-13,23)	0.244
HDL-cholesterol (mg/dL) ₀	55(45,67)a	46(41,55)b	51(43,58)a	49(43,59)b	0.002
Δ HDL-cholesterol (mg/dL)	-1(-7,5)	-2(-8,2)	-2(-8,3)	-2(-7,2)	0.727
Triglyceride (mg/dL) ₀	109(88,144)a	125(98,175)b	121(91,155)a	144(103,188)b	0.001
Δ Triglyceride (mg/dL) ₀	6(-21,34)a	16(-5,44)b	7(-17,29)a	19(-15,56)b	0.042
TG / HDL ratio ₀	1.92(1.29,3.1)a	2.76(2,4.3)b	2.36(1.66,3.28)a	2.81(1.82,4.13)b	<.001
Δ TG / HDL ratio	0.19(-0.35,0.64)	0.35(-0.13,1.43)	0.12(-0.4,0.86)	0.43(-0.29,1.44)	0.075
Glucose (mg/dL)	88(79,97)	89(78,95)	87(80,97)	89(78,96)	0.961
Δ Glucose (mg/dL)	-6(-17,3)	-7(-15,-1)	-8(-16,2)	-6(-18,1)	0.694
High-sensitivity CRP (mg/dL) ₀	0.3(0.2,0.6)a	0.3(0.2,0.6)a	0.4(0.3,1)a,b	0.6(0.3,1.4)b	0.002
Δ High-sensitivity CRP (mg/dL)	-0.2(-0.6,0)	-0.1(-0.5,0.1)	-0.1(-0.4,0.1)	-0.1(-0.5,0.1)	0.535

Data are presented as the mean ± standard deviation or (25 percentile, 75 percentile) for continuous variables and the number (%) for categorical variables.

Δ, variation from baseline; BMI, body mass index; LDL, low-density lipoprotein; HDL, high-density lipoprotein; TG/HDL, Triglyceride/HDL-cholesterol; CRP, C-reactive protein.

^₀^ Multiple comparison (post-hoc) results, same letter means no difference.

Although Group 2 had low serum adiponectin level and Group 3 had high VFA, Table 2 showed the similar results between Group 2 and Group 3. Further comparison between Group 2 and Group 3 was showed in the S1 Table. The most significant differences between Group 2 and Group 3 were waist circumference and body mass index. Group 3 had larger waist circumference and BMI compared to Group 2 (waist circumference; 85 vs 89.5 cm, BMI; 23.37 vs 25.16 kg/m2, both p <.001). Other blood pressure, lipid profile, glucose and high-sensitivity CRP were similar except that the follow-up HDL-cholesterol were lower (46 vs 51 mg/dL, p = 0.049) and the increase of triglyceride level was bigger (16 vs 7 mg/dL, p = 0.024) in Group 2 compared to Group 3.

For the further delineation of the spectrum between higher VFA and lower serum adiponectin level, we analyzed the changes of metabolic syndrome risk factors dependent to the tertile of VFA and the tertile of serum adiponectin level (S2 and S3 Tables). Both higher VFA and lower serum adiponectin level showed significant unfavorable effect with the metabolic syndrome risk factors at the follow-up (waist circumference, BMI, systolic and diastolic blood pressure, HDL-cholesterol, triglyceride and triglyceride/HDL-cholesterol ratio). High-sensitivity CRP level has greater value in the higher VFA. Interestingly, VFA was associated with waist circumference change, and serum adiponectin level was associated with the changes of triglyceride and the triglyceride/HDL-cholesterol ratio. Those data suggested that adiponectin and VFA might contribute to metabolic syndrome with somewhat different mechanisms although adiponectin and VFA were significantly associated with each other.

### Risk of metabolic syndrome and its components

The effects of adiponectin level and VFA at baseline were analyzed using multivariable-adjusted models of metabolic syndrome and its five components (Table 3). Model 1 included age and sex. Model 2 included the lifestyle-related factors (smoking, alcohol, education level and physical activity). Model 3 included the 5 metabolic syndrome risk factors (waist circumference, triglyceride, HDL-cholesterol, blood pressure, and glucose). Model 4 included the other precise biomarkers (high sensitivity CRP, LDL-cholesterol, apolipoprotein AI, and apolipoprotein B). In Model 4, in which all the factors were adjusted, higher VFA was significantly associated with the development of metabolic syndrome (T3 vs T1; OR, 2.920; 95% CI, 1.101–7.747; p = 0.031). Among the metabolic syndrome risk factors, high waist circumference was significantly associated with higher VFA (OR, 4.188; 95% CI, 1.528–11.479; p = 0.005). Serum adiponectin level was also significantly associated with the development of metabolic syndrome(OR, 0.419; 95% CI, 0.199–0.884; p = 0.022). The combination of higher VFA and lower adiponectin levels (Groups 4 vs 1) was also demonstrated to be significantly associated with the development of metabolic syndrome (OR, 4.918; 95% CI, 2.05–11.795; p <.001). Among the metabolic syndrome risk factors, high waist circumference and high triglyceride levels were significantly associated with Group 4. For high waist circumference, OR was 2.827 (95% CI, 1.154–6.925; p = 0.026) and for high triglyceride, OR was 3.508 (95% CI, 1.537–8.007; p = 0.007). Subgroup analysis showed that the disfavoring effect of Group 4 for metabolic syndrome appeared consistent across sex (S4 Table).

**Table 3:** Associations among adiponectin, visceral fat area and their groups for metabolic syndrome and its risk factors.

		Adiponectin (T3 vs T1)	Visceral fat area (T3 vs T1)	Group (Group 4 vs Group 1)
Metabolic syndrome	Crude Model	0.545(0.305,0.972)	1.725(0.924,3.218)	4.347(2.146,8.806)
	Model 1	0.365(0.188,0.706)	2.661(1.307,5.417)	4.464(2.193,9.088)
	Model 2	0.324(0.159,0.659)	3.392(1.565,7.354)	5.907(2.753,12.676)
	Model 3	0.426(0.203,0.893)	2.883(1.107,7.507)	4.72(2.012,11.072)
	Model 4	0.419(0.199,0.884)	2.92(1.101,7.747)	4.918(2.05,11.795)
High waist circumference	Crude Model	0.662(0.389,1.126)	7.381(4.089,13.324)	5.463(2.846,10.488)
	Model 1	0.688(0.383,1.236)	10.816(5.361,21.823)	5.513(2.862,10.619)
	Model 2	0.677(0.367,1.249)	16.745(7.678,36.52)	6.584(3.284,13.2)
	Model 3	0.661(0.288,1.517)	4.123(1.542,11.021)	2.717(1.14,6.477)
	Model 4	0.623(0.269,1.447)	4.188(1.528,11.479)	2.827(1.154,6.925)
High blood pressure	Crude Model	0.694(0.401,1.2)	1.371(0.801,2.345)	1.104(0.591,2.061)
	Model 1	0.933(0.509,1.712)	1.011(0.547,1.869)	1.117(0.594,2.099)
	Model 2	0.97(0.515,1.829)	1.026(0.534,1.972)	1.15(0.596,2.221)
	Model 3	0.901(0.434,1.87)	1.159(0.468,2.867)	1.237(0.552,2.77)
	Model 4	0.896(0.431,1.865)	1.155(0.456,2.927)	1.252(0.547,2.868)
High glucose	Crude Model	0.848(0.428,1.681)	0.899(0.444,1.818)	0.677(0.313,1.463)
	Model 1	1.015(0.476,2.166)	0.648(0.291,1.44)	0.672(0.31,1.458)
	Model 2	1.072(0.479,2.396)	0.784(0.334,1.841)	0.691(0.305,1.566)
	Model 3	1.438(0.493,4.194)	0.659(0.189,2.294)	0.516(0.157,1.691)
	Model 4	1.441(0.488,4.255)	0.665(0.188,2.344)	0.527(0.158,1.755)
High triglyceride	Crude Model	0.56(0.327,0.959)	1.492(0.867,2.57)	2.619(1.415,4.848)
	Model 1	0.544(0.301,0.985)	1.511(0.818,2.79)	2.659(1.434,4.932)
	Model 2	0.507(0.271,0.951)	1.46(0.757,2.814)	3.06(1.593,5.875)
	Model 3	0.584(0.291,1.172)	1.461(0.618,3.452)	3.649(1.621,8.215)
	Model 4	0.601(0.297,1.217)	1.328(0.555,3.176)	3.508(1.537,8.007)
Low HDL-cholesterol	Crude Model	1.147(0.666,1.978)	0.634(0.362,1.109)	1.611(0.869,2.986)
	Model 1	0.659(0.355,1.224)	1.004(0.531,1.901)	1.628(0.866,3.06)
	Model 2	0.642(0.33,1.249)	1.124(0.562,2.247)	1.788(0.919,3.482)
	Model 3	0.814(0.382,1.738)	1.665(0.638,4.344)	1.962(0.84,4.583)
	Model 4	0.807(0.374,1.743)	1.831(0.689,4.866)	2.024(0.847,4.833)

Data are presented as odd ratio (95% confidence intervals).

Model 1. Adjusted variable: age, sex.

Model 2. Adjusted variable: Model 1 + smoking, alcohol, education level, physical activity.

Model 3. Adjusted variable: Model 2 + waist circumference, triglyceride, HDL-cholesterol, systolic blood pressure, diastolic blood pressure, glucose.

Model 4. Adjusted variable: Model 3 + high sensitivity CRP, LDL-cholesterol, apolipoprotein AI, apolipoprotein B.

## Discussion

The main finding of the present study was that subjects with higher VFA and lower serum adiponectin levels exhibited a significantly higher risk for the development of metabolic syndrome. Notably, the combination of higher VFA and lower serum adiponectin levels was an independent predictor for metabolic syndrome following adjustment for the other important risk factors including waist circumference, blood pressure, glucose, lipid profile, and other biomarkers.

Previously, visceral fat has been shown to be associated with a greater cardiometabolic risk, as compared with subcutaneous fat [28] and other obesity measurements, including BMI [12]. The present study demonstrated that the highest quartiles of visceral adipose tissue exhibited a higher risk of metabolic syndrome, as compared with the lowest quartiles after covariate adjustments (Table 3). Subgroup analyses of the metabolic syndrome component also indicated that visceral fat was independently associated with waist circumference rather than the other metabolic syndrome components. Therefore, increased VFA potentiated the risk of metabolic syndrome predominantly driven by a higher waist circumference.

Several studies have demonstrated that serum adiponectin levels are inversely correlated with visceral adipose tissue [29]. Baseline characteristics in the present study also showed the inverse relationship between VFA and serum adiponectin levels in the community-based general population without metabolic syndrome (S1 Fig). Notably, these associations were differently affected by sex. Women exhibited reduced VFA and increased serum adiponectin levels compared to men (p <.001). Furthermore, women had a strong negative correlation between VFA and serum adiponectin levels (r = -0.328, p <.001); whereas men had a weak positive correlation without statistical significance (r = 0.071, p = 0.391). These findings were similar to the results from the western country [30].

Previous studies reported that serum adiponectin was decreased in obese subjects and associated with metabolic syndrome components [31]. High serum adiponectin levels have also been reported to be associated with reduced insulin resistance and other favorable effects including anti-inflammatory and anti-atherogenic properties [32]. In the present study, serum adiponectin level exhibited a significant association with metabolic syndrome development. Although there was no statistical significance, high triglyceride showed the lowest odd ratio among 5 metabolic syndrome risk factors. There may be several explanations for this. First, the effect of serum adiponectin levels may be potentiated in a manner that is dependent on visceral adiposity. Adiponectin levels have been reported to increase or remain constant with aging [33,34]. Recently, Li JB et al demonstrated that adiponectin levels and the visceral fat ratio decreased with aging in an animal model [35]. This finding suggested that relatively insufficient amounts of adiponectin on visceral adipose tissue may be associated with an increased risk of the progression of metabolic syndrome. Second, there might be some functional differences among the adiponectins secreted from different adipose tissues. Adiponectin circulates in different multimeric forms [36]. High molecular weight adiponectin exhibited a greater association with insulin sensitivity, as compared with total adiponectin [37]. Different forms of adiponectin have been reported to exhibit binding properties with other proteins including C1q, and these protein complexes may reflect the risk of metabolic syndrome [38]. Notably, visceral adipose tissue has been associated with C1q-adiponecitn complex levels.

The present study has some limitations. Although this study was based on a community-based cohort, it was performed in a single center and the follow-up loss rate was up to 33.1%. Therefore, our study population may not represent the whole general population in the community, and the selection bias may limit our interpretation. Moreover, the small sample size and the short follow-up duration (median 17 months) also abate the precision of risk estimation in the present study. In addition, we only evaluated a single measurement of serum adiponectin levels and VFA at baseline. Follow-up measurements may explain the mechanism of their independent roles for the development of metabolic syndrome. Despite these limitations, the major strength of the present study is that the data were collected from a community-based cohort study. The present findings may contribute to the establishment of causality between the indices (visceral fat and adiponectin) and metabolic syndrome.

In addition, considering that the prediction and prevention of metabolic syndrome are now considered of great importance for public health, our findings may contribute to assess the high risk population for metabolic syndrome. A future intervention trials should evaluate the benefits of a screening the high risk group for metabolic syndrome.

## Conclusion

In a community-based population without overt metabolic risks, the combination of low serum adiponectin levels and high VFA significantly predicted the development of metabolic syndrome, which was predominantly driven by an increase in triglyceride level. Higher insulin resistance and systemic inflammation, indicated by a higher triglyceride to HDL cholesterol ratio and higher hsCRP level, may also contribute to the development of metabolic syndrome in subjects with low serum adiponectin levels and high VFA.

## Supporting Information

S1 Fig Correlation between adiponectin and visceral fat area. (TIF)

S1 Table Comparison of the follow-up characteristics and their respective variations from the baseline between Group 2 and Group 3. Data are presented as the mean ± standard deviation or (25 percentile, 75 percentile) for continuous variables and the number (%) for categorical variables. Δ, variation from baseline; BMI, body mass index; LDL, low-density lipoprotein; HDL, high-density lipoprotein; TG/HDL, Triglyceride/HDL-cholesterol; CRP, C-reactive protein.
(DOCX)

S2 Table Comparison of the follow-up characteristics and their respective variations from the baseline according to the visceral fat area tertile groups. Data are presented as the mean ± standard deviation or (25 percentile, 75 percentile) for continuous variables and the number (%) for categorical variables. Δ, variation from baseline; BMI, body mass index; LDL, low-density lipoprotein; HDL, high-density lipoprotein; TG/HDL, Triglyceride/HDL-cholesterol; CRP, C-reactive protein.
(DOCX)

S3 Table Comparison of the follow-up characteristics and their respective variations from the baseline according to the serum adiponectin level tertile groups. Data are presented as the mean ± standard deviation or (25 percentile, 75 percentile) for continuous variables and the number (%) for categorical variables. Δ, variation from baseline; BMI, body mass index; LDL, low-density lipoprotein; HDL, high-density lipoprotein; TG/HDL, Triglyceride/HDL-cholesterol; CRP, C-reactive protein.
(DOCX)

S4 Table Effect of sex on the association between serum adiponectin/visceral fat area groups and the incidence of metabolic syndrome. Data was presented as odd ratio (95% confidence interval). OR, odd ratio; 95% CI, 95% confidence interval.
(DOCX)
