# Supplementary figures and images for: Visceral Fat Area and Serum Adiponectin Level Predict the Development of Metabolic Syndrome in a Community-Based Asymptomatic Population
Source: PLoS One. 2017 Jan 3;12(1):e0169289. doi: 10.1371/journal.pone.0169289 (PMC5207404; doi:10.1371/journal.pone.0169289)

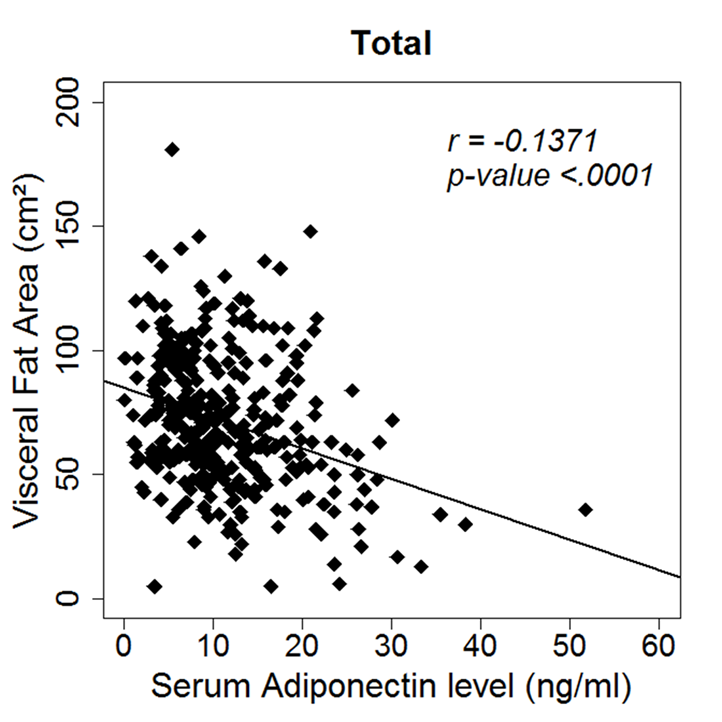

Supplement: S1 Fig — (TIF) [file pone.0169289.s001.tif]
