# Supplementary material for: Visceral Fat Area and Serum Adiponectin Level Predict the Development of Metabolic Syndrome in a Community-Based Asymptomatic Population
Source: PLoS One. 2017 Jan 3;12(1):e0169289. doi: 10.1371/journal.pone.0169289 (PMC5207404; doi:10.1371/journal.pone.0169289)
Supplement: S1 Table — Data are presented as the mean ± standard deviation or (25 percentile, 75 percentile) for continuous variables and the number (%) for categorical variables. Δ, variation from baseline; BMI, body mass index; LDL, low-density lipoprotein; HDL, high-density lipoprotein; TG/HDL, Triglyceride/HDL-cholesterol; CRP, C-reactive protein. (DOCX) [file pone.0169289.s002.docx]

**S1 Table. Comparison of the follow-up characteristics and their respective variations from the baseline between Group 2 and Group 3.**

| **Variable** | **Group 2 (N=77)** | **Group 3 (N=84)** | **p-value** |
| --- | --- | --- | --- |
| Waist circumference (cm) | 85(81,89) | 89.5(85,94.5) | <.001 |
| Δ Waist circumference (cm) | 1.5(0,5) | 1(-1,3.5) | 0.239 |
| BMI (kg/m2) | 23.37(22.06,24.8) | 25.16(23.94,27.54) | <.001 |
| Δ BMI (kg/m2) | -0.3(-0.97,0.32) | -0.32(-0.95,0.36) | 0.922 |
| Systolic blood pressure (mmHg) | 120.7±13.06 | 119.2±14.56 | 0.494 |
| Δ Systolic blood pressure (mmHg) | 2.75±12.67 | 1.63±12.77 | 0.512 |
| Diastolic blood pressure (mmHg) | 80.77±8.54 | 78.65±10.41 | 0.161 |
| Δ Diastolic blood pressure (mmHg) | 6.48±8.07 | 5.68±7.32 | 0.503 |
| Pulse pressure (mmHg) | 72(66,84) | 70.75(62,76.75) | 0.072 |
| Δ Pulse pressure (mmHg) | 2.5(-3,7.5) | 0(-6.5,6) | 0.121 |
| Total Cholesterol (mg/dL) | 199(180,219) | 200(183,226) | 0.661 |
| Δ Total Cholesterol (mg/dL) | 8(-9,27) | 2(-19,15) | 0.077 |
| LDL-cholesterol (mg/dL) | 135.1±32.07 | 136.25±28.78 | 0.812 |
| Δ LDL-cholesterol (mg/dL) | 3(-11,17) | 1(-15,16) | 0.538 |
| HDL-cholesterol (mg/dL) | 46(41,55) | 51(43,58) | 0.049 |
| Δ HDL-cholesterol (mg/dL) | -2(-8,2) | -2(-8,3) | 0.791 |
| Triglyceride (mg/dL) | 125(98,175) | 121(91,155) | 0.161 |
| Δ Triglyceride (mg/dL) | 16(-5,44) | 7(-17,29) | 0.024 |
| TG / HDL ratio | 2.76(2,4.3) | 2.36(1.66,3.28) | 0.082 |
| Δ TG / HDL ratio | 0.35(-0.13,1.43) | 0.12(-0.4,0.86) | 0.065 |
| Glucose (mg/dL) | 89(78,95) | 87(80,97) | 0.683 |
| Δ Glucose (mg/dL) | -7(-15,-1) | -8(-16,2) | 0.696 |
| High-sensitivity CRP (mg/dL) | 0.3(0.2,0.6) | 0.4(0.3,1)a | 0.053 |
| Δ High-sensitivity CRP (mg/dL) | -0.1(-0.5,0.1) | -0.1(-0.4,0.1) | 0.803 |

Data are presented as the mean ± standard deviation or (25 percentile, 75 percentile) for continuous variables and the number (%) for categorical variables.

Δ, variation from baseline; BMI, body mass index; LDL, low-density lipoprotein; HDL, high-density lipoprotein; TG/HDL, Triglyceride/HDL-cholesterol; CRP, C-reactive protein.
