# Supplementary material for: Visceral Fat Area and Serum Adiponectin Level Predict the Development of Metabolic Syndrome in a Community-Based Asymptomatic Population
Source: PLoS One. 2017 Jan 3;12(1):e0169289. doi: 10.1371/journal.pone.0169289 (PMC5207404; doi:10.1371/journal.pone.0169289)
Supplement: S2 Table — Data are presented as the mean ± standard deviation or (25 percentile, 75 percentile) for continuous variables and the number (%) for categorical variables. Δ, variation from baseline; BMI, body mass index; LDL, low-density lipoprotein; HDL, high-density lipoprotein; TG/HDL, Triglyceride/HDL-cholesterol; CRP, C-reactive protein. (DOCX) [file pone.0169289.s003.docx]

S**2 Table. Comparison of the follow-up characteristics and their respective variations from the baseline according to the visceral fat area tertile groups.**

| **Variable** | **VFA < 58 cm^2^** | **VFA 58 – 79 cm^2^** | **VFA ≥ 79 cm^2^** | **p-value** | **p_trend_-value** |
| --- | --- | --- | --- | --- | --- |
| Waist circumference (cm) | 83(78.5,87) | 87(83,91) | 92(88,96) | <.0001 | <.0001 |
| Δ Waist circumference (cm) | 3(-1,5) | 2(-1,5) | 0.5(-2,3.5) | 0.003 | 0.0017 |
| BMI (kg/m2) | 23.14(21.49,24.38) | 24.07(23.23,25.64) | 26.35(25.01,28.25) | <.0001 | <.0001 |
| Δ BMI (kg/m2) | -0.28(-0.93,0.29) | -0.31(-1.08,0.28) | -0.33(-0.78,0.38) | 0.619 | 0.7581 |
| Systolic blood pressure (mmHg) | 119.28±13.46 | 119.02±13.63 | 122.87±13.29 | 0.0564 | 0.0458 |
| Δ Systolic blood pressure (mmHg) | 1.85±13.95 | 2.42±12.27 | 1.21±12.39 | 0.7785 | 0.7075 |
| Diastolic blood pressure (mmHg) | 78.68±8.65 | 77.91±8.87 | 82.03±8.75 | 0.0009 | 0.0042 |
| Δ Diastolic blood pressure (mmHg) | 5.05±7.05 | 5.98±8.27 | 5.64±7.33 | 0.6532 | 0.5583 |
| Pulse pressure (mmHg) | 71(63.5,80.5) | 72(65.5,78.5) | 70.5(64,79.5) | 0.6706 | 0.8178 |
| Δ Pulse pressure (mmHg) | 0(-7,6.5) | 1.75(-6.5,8) | 0(-7,5.5) | 0.645 | 0.6293 |
| Total Cholesterol (mg/dL) | 207(185,231) | 207(183,236) | 199(185,224) | 0.5808 | 0.4303 |
| Δ Total Cholesterol (mg/dL) | 9(-10,28) | 7(-14,25) | 0(-12,16) | 0.5345 | 0.2634 |
| LDL-cholesterol (mg/dL) | 139.86±31.79 | 139.05±36.98 | 134.5±28.64 | 0.4124 | 0.2182 |
| Δ LDL-cholesterol (mg/dL) | 6(-10,27) | 4(-16,23) | 1(-12,17) | 0.3525 | 0.1493 |
| HDL-cholesterol (mg/dL) | 52(44,64) | 49(42,59) | 48(42,58) | 0.0075 | 0.0021 |
| Δ HDL-cholesterol (mg/dL) | -2(-9,5) | -2(-8,3) | -1(-6,2) | 0.7992 | 0.5512 |
| Triglyceride (mg/dL) | 108(86,144) | 138(102,175) | 134(102,180) | 0.0058 | 0.0034 |
| Δ Triglyceride (mg/dL) | 9(-10,40) | 13(-21,36) | 9(-17,39) | 0.5293 | 0.781 |
| TG / HDL ratio ₀ | 1.94(1.29,3.33) | 2.76(1.86,4.09) | 2.63(1.83,3.61) | 0.0123 | 0.0053 |
| Δ TG / HDL ratio | 0.21(-0.26,0.9) | 0.33(-0.42,1.06) | 0.39(-0.31,0.97) | 0.4236 | 0.6852 |
| Glucose (mg/dL) | 87(79,96) | 89(78,97) | 89(79,95) | 0.6137 | 0.8849 |
| Δ Glucose (mg/dL) | -6(-13,1) | -7(-18,1) | -7(-18,2) | 0.6066 | 0.325 |
| High-sensitivity CRP (mg/dL) ₀ | 0.3(0.2,0.5) | 0.4(0.2,0.9) | 0.6(0.3,1.5) | 0.0168 | 0.0156 |
| Δ High-sensitivity CRP (mg/dL) | -0.1(-0.4,0) | -0.1(-0.5,0.1) | -0.1(-0.5,0.2) | 0.2815 | 0.1122 |

Data are presented as the mean ± standard deviation or (25 percentile, 75 percentile) for continuous variables and the number (%) for categorical variables.

Δ, variation from baseline; BMI, body mass index; LDL, low-density lipoprotein; HDL, high-density lipoprotein; TG/HDL, Triglyceride/HDL-cholesterol; CRP, C-reactive protein.
