# Supplementary material for: Visceral Fat Area and Serum Adiponectin Level Predict the Development of Metabolic Syndrome in a Community-Based Asymptomatic Population
Source: PLoS One. 2017 Jan 3;12(1):e0169289. doi: 10.1371/journal.pone.0169289 (PMC5207404; doi:10.1371/journal.pone.0169289)
Supplement: S3 Table — Data are presented as the mean ± standard deviation or (25 percentile, 75 percentile) for continuous variables and the number (%) for categorical variables. Δ, variation from baseline; BMI, body mass index; LDL, low-density lipoprotein; HDL, high-density lipoprotein; TG/HDL, Triglyceride/HDL-cholesterol; CRP, C-reactive protein. (DOCX) [file pone.0169289.s004.docx]

**S3 Table. Comparison of the follow-up characteristics and their respective variations from the baseline according to the serum adiponectin level tertile groups.**

| **Variable** | **Adiponectin**  **< 7.5 ng/mL** | **Adiponectin**  **7.5 – 13.0 cm^2^** | **Adiponectin**  **> 13.0 cm^2^** | **p-value** | **p_trend_-value** |
| --- | --- | --- | --- | --- | --- |
| Waist circumference (cm) | 90(86,93.5) | 86(82,90) | 85.5(81,92) | <.0001 | 0.0002 |
| Δ Waist circumference (cm) | 2(-1,5) | 1(-1,4) | 2(-1,5) | 0.0947 | 0.3717 |
| BMI (kg/m2) | 25.61(23.44,27.02) | 23.94(22.89,25.64) | 24.03(22.58,26.64) | 0.0058 | 0.0131 |
| Δ BMI (kg/m2) | -0.32(-0.93,0.32) | -0.39(-1.05,0.27) | -0.15(-0.9,0.37) | 0.2799 | 0.4348 |
| Systolic blood pressure (mmHg) | 122.72±12.91 | 120.75±12.89 | 117.87±14.39 | 0.0246 | 0.0069 |
| Δ Systolic blood pressure (mmHg) | 2.29±12.2 | 1.72±12.55 | 1.46±13.84 | 0.8864 | 0.6311 |
| Diastolic blood pressure (mmHg) | 81.2±8.24 | 80.37±8.45 | 77.23±9.55 | 0.0018 | 0.0008 |
| Δ Diastolic blood pressure (mmHg) | 5.85±8.39 | 6.11±7.46 | 4.72±6.73 | 0.3404 | 0.2647 |
| Pulse pressure (mmHg) | 72(67,81.5) | 70.5(64,79.5) | 71.5(62.5,78) | 0.293 | 0.1174 |
| Δ Pulse pressure (mmHg) | 0.5(-7,6.5) | 1(-5,7.5) | -1(-8.5,6) | 0.1251 | 0.092 |
| Total Cholesterol (mg/dL) | 199(183,227) | 201.5(181,226) | 209(187,232) | 0.7439 | 0.9171 |
| Δ Total Cholesterol (mg/dL) | 9(-9,28) | 0(-11,19) | 8(-13,23) | 0.2201 | 0.2079 |
| LDL-cholesterol (mg/dL) | 136.23±32.63 | 137.29±32.14 | 139.73±33.2 | 0.7109 | 0.4218 |
| Δ LDL-cholesterol (mg/dL) | 5(-11,22) | -1(-13,15) | 8(-12,24) | 0.8175 | 0.8039 |
| HDL-cholesterol (mg/dL) | 47(42,56) | 50.5(40,59) | 54(45,64) | 0.0018 | 0.0004 |
| Δ HDL-cholesterol (mg/dL) | -1(-6,2) | -2.5(-8,3) | -2(-9,5) | 0.9533 | 0.8946 |
| Triglyceride (mg/dL) | 135(106,186) | 124.5(94,178) | 111(87,148) | 0.0001 | <.0001 |
| Δ Triglyceride (mg/dL) | 23(-8,57) | 9(-17,34) | 5(-17,31) | 0.0698 | 0.0281 |
| TG / HDL ratio ₀ | 2.81(2.13,4.24) | 2.55(1.63,4.08) | 2.15(1.42,3.1) | <.0001 | <.0001 |
| Δ TG / HDL ratio | 0.43(-0.2,1.43) | 0.21(-0.32,0.82) | 0.2(-0.35,0.8) | 0.1425 | 0.0491 |
| Glucose (mg/dL) | 89(79,95) | 87.5(76,97) | 88(81,95) | 0.3817 | 0.5715 |
| Δ Glucose (mg/dL) | -6(-15,1) | -10.5(-19,-1) | -6(-13,2) | 0.0488 | 0.7373 |
| High-sensitivity CRP (mg/dL) ₀ | 0.4(0.2,1.2) | 0.5(0.2,1.2) | 0.4(0.2,0.7) | 0.21 | 0.0791 |
| Δ High-sensitivity CRP (mg/dL) | -0.1(-0.6,0.1) | -0.1(-0.4,0.2) | -0.1(-0.5,0.1) | 0.7665 | 0.4727 |

Data are presented as the mean ± standard deviation or (25 percentile, 75 percentile) for continuous variables and the number (%) for categorical variables.

Δ, variation from baseline; BMI, body mass index; LDL, low-density lipoprotein; HDL, high-density lipoprotein; TG/HDL, Triglyceride/HDL-cholesterol; CRP, C-reactive protein.
