# Supplementary material for: Visceral Fat Area and Serum Adiponectin Level Predict the Development of Metabolic Syndrome in a Community-Based Asymptomatic Population
Source: PLoS One. 2017 Jan 3;12(1):e0169289. doi: 10.1371/journal.pone.0169289 (PMC5207404; doi:10.1371/journal.pone.0169289)
Supplement: S4 Table — Data was presented as odd ratio (95% confidence interval). OR, odd ratio; 95% CI, 95% confidence interval. (DOCX) [file pone.0169289.s005.docx]

**S4 Table. Effect of sex on the association between serum adiponectin/visceral fat area groups and the incidence of metabolic syndrome.**

|  | **Group** | **OR (95%CI)** | **p-value** | **p_interaction_-value** |
| --- | --- | --- | --- | --- |
| Men | Group 1 | 1 | - | 0.6418 |
|  | Group 2 | 2.131 (0.413 - 11.006) | 0.3665 |  |
|  | Group 3 | 2.19 (0.407 - 11.801) | 0.3615 |  |
|  | Group 4 | 5.057 (1.096 - 23.346) | 0.0378 |  |
| Women | Group 1 | 1 | - |  |
|  | Group 2 | 0.718 (0.193 - 2.673) | 0.6211 |  |
|  | Group 3 | 1.899 (0.478 - 7.548) | 0.3622 |  |
|  | Group 4 | 4.152 (1.103 - 15.631) | 0.0353 |  |

Data was presented as odd ratio (95% confidence interval).

OR, odd ratio; 95% CI, 95% confidence interval
